# Centromeres drive and take a break

**DOI:** 10.1007/s10577-025-09777-z

**Published:** 2025-08-04

**Authors:** Paul B. Talbert, Steven Henikoff

**Affiliations:** Howard Hughes Medical Institute, Fred Hutch Cancer Center, 1100 Fairview Avenue N, Seattle, WA 98109 USA

**Keywords:** Kinetochore, Satellite DNA, Centromere drive, Break-induced replication, Speciation, Cancer

## Abstract

The identification of CENPA, CENPB, and CENPC by Earnshaw and Rothfield 40 years ago has revealed the remarkable diversity and complexity of centromeres and confirmed most seed plants and animals have centromeres comprised of complex satellite arrays. The rapid evolution of centromeres and positive selection on CENPA and CENPC led to the centromere drive model, in which competition between tandem satellite arrays of differing size and centromere strength for inclusion in the egg of animals or megaspore of seed plants during female meiosis drives rapid evolution of centromeres and kinetochore proteins. Here we review recent work showing that non-B-form DNA structures in satellite centromeres make them sites of frequent replication fork stalling, and that repair of collapsed forks by break-induced replication rather than unequal sister chromatid exchange is likely the primary mode of satellite expansion and contraction, providing the variation in satellite copy number that is the raw material of centromere drive. Centromere breaks at replication, rather than errors at mitosis, can account for most centromere misdivisions that underlie aneuploidies in cancer.

## Introduction: The ABCs of kinetochores

Fifty years after Walther Flemming described the “primary constriction” that mediates spindle attachment to chromosomes during mitosis (Flemming 1882), Barbara McClintock discovered that this “insertion region” can be broken into two functional parts that can each attach to the spindle (McClintock 1932), and C.D. Darlington proposed that such misdivision of the “centromere” implied that it was composed of multiple subunits that he called “centrogenes” (Darlington 1939). In another 50 years, the first molecular characterization of the DNA of a centromere was carried out in budding yeast (Fitzgerald-Hayes et al. 1982). Budding yeast centromeres were found to have ~ 88 bp AT-rich sequences flanked by short conserved sites together totaling ~ 125 bp, and lacked multiple copies of any apparent “centrogenes”. Only three years later, Earnshaw and Rothfield, using antibodies from pre-immune serum of CREST (calcinosis, Raynaud’s phenomenon, esophageal dysmotility, sclerodactyly, telangiectasia) patients, identified three centromere protein components (CENPs) found at centromeres (i.e. kinetochore proteins), which they named CENPA, CENPB, and CENPC (Earnshaw & Rothfield 1985). They observed that on mitotic chromosomes from HeLa cells, centromeres that were stretched could be seen to have two half-kinetochores, an observation that Earnshaw’s group followed up in molecular detail nearly 40 years later (Sacristan et al. 2024). Antibodies to these proteins, as well as tagged constructs of these proteins, have been invaluable tools in the study of centromeres and kinetochores over the past 40 years, and revealed a great diversity in centromere structure and size across eukaryotes from 125 bp “point centromeres” of budding yeast to holocentromeres encompassing the lengths of chromosomes in several lineages of flowering plants and animals, reviewed in (Talbert & Henikoff 2020). In this review we will focus on the properties of one structural class of centromeres, the satellite centromeres.

At the time of Earnshaw and Rothfield’s discovery, it was known that in animals centromere regions and pericentric heterochromatin were largely occupied by highly tandemly repeated short sequences, known as satellites because of their biased, usually AT-rich composition and consequent different buoyant density than bulk DNA in a CsCl gradient (Kit 1961, 1962). Their tandem repetitiveness is suggestive of Darlington’s centrogenes. Satellites did not code for protein, differed between related organisms, evolved rapidly, and were hypothesized to be involved in speciation, reviewed in (Yunis & Yasmineh 1971). It was becoming apparent that unlike the ~ 125 bp centromeres of budding yeast, human centromeres likely encompass related families of highly tandemly repeated 171 bp non-coding sequences known as α-satellites, that are both species- and chromosome-specific (Waye & Willard 1989; Willard 1985). Soon CREST anti-kinetochore serum was shown to bind antigens that co-localize with α-satellite (Masumoto et al. 1989a, b) and CENPB was found to bind to a 17 bp motif (CENPB box) present in a subset of α-satellite monomers (Masumoto et al. 1989a, b). CENPA was revealed to be a centromere-specific variant of histone H3, for which it could substitute in nucleosomes (Palmer et al. 1991, 1987; Sullivan et al. 1994), and its role as a foundational component of the inner kinetochore was confirmed by immunoprecipitating α-satellite DNA bound by a tagged version of CENPA (Vafa & Sullivan 1997). Similarly, CENPC was likewise shown to be an inner kinetochore protein that binds α-satellite DNA preferentially in vivo*,* though it binds DNA non-specifically in vitro*.* (Politi et al. 2002; Saitoh et al. 1992; Sugimoto et al. 1994; Yang et al. 1996). Minichromosomes that bound CENPB, CENPC, and the spindle kinesin CENPE were produced by amplification of homogeneous HORs (higher order repeats) of α-satellite containing CENPB boxes, but not by more divergent α-satellite, suggesting that specific α-satellite subfamilies specified the centromere (Harrington et al. 1997; Ikeno et al. 1998). However, as it became clear that α-satellite comprised human centromeres, it also became clear that, except for the 125 bp centromeres of budding yeast and relatives, centromere function was primarily epigenetic (Murphy & Karpen 1998). Human dicentric chromosomes were recovered in which one α-satellite array bound CENPC and CENPE while the other did not, while both bound CENPB (Sullivan & Schwartz 1995). Human marker chromosomes were described that assembled CENPA and CENPC on sequences that had not previously been centromeric (neocentromeres) and that lacked α-satellite (Amor & Choo 2002; du Sart et al. 1997). If satellite sequences are neither necessary nor sufficient for centromere formation, why are they commonly found abundantly at animal and plant centromeres?

## Satellites organize CENPA nucleosomes

Centromeric satellite monomers come in many lengths, but are most frequently 100–400 bp, the approximate length of one or two nucleosomes (Melters et al. 2013), suggesting an organizational relationship to centromeric nucleosomes. The identification of CENPA permitted the identification of centromere-specific H3 variants in most eukaryotes (van Hooff et al. 2017). In plants the centromere-specific H3 variant corresponding to CENPA is usually known as CENH3 (Talbert et al. 2002; Zhong et al. 2002). Centromeric satellite arrays can impose translational and rotational phasing on CENPA/CENH3 nucleosomes so that they are regularly spaced and interact with the same “face” of the double helix in each satellite monomer (Hasson et al. 2013; Huang et al. 2021; Iwata-Otsubo et al. 2017; Naish et al. 2021; Su et al. 2019; Yang et al. 2018; Zhang et al. 2013). A single turn of the DNA double helix is ~ 10 bp, and the 10 bp periodicity of WW dinucleotides (W = A or T), especially AA dinucleotides, found in many centromeric satellites (Talbert & Henikoff 2020) favors nucleosome formation by minimizing bending energy to wrap DNA around a nucleosome (Prytkova et al. 2011; Struhl & Segal 2013). This suggests that satellite arrays may stabilize CENPA/CENH3 nucleosomes against the pulling forces of anaphase and/or provide a favorable organizational template for kinetochore formation.

**Fig. 1:**
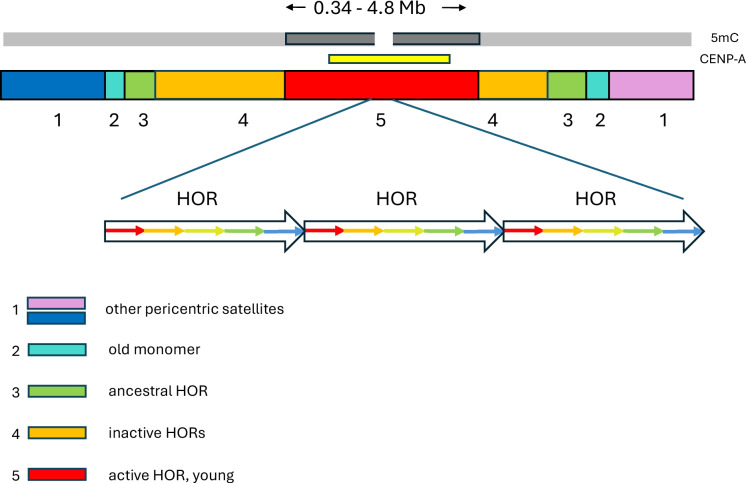
**Model of human centromere** Centromeres have a layered structure with the youngest active HOR flanked by older, more diverse HORs flanked by α-satellite monomers and other types of pericentric satellites. CENP-A is generally confined to the active HOR, including a centromere dip region of reduced CG DNA methylation (5mC). HORs are higher order repeats of sets of more diverged monomers

In humans centromeric α-satellites form HORs, in which the individual monomers of a HOR are diverged from each other but the copies of the HOR are nearly identical (Logsdon & Eichler 2022). There may be multiple HORs on a chromosome but only one HOR of 2 or 5 monomers, depending on the chromosome, forms the active centromere. Centromeric α-satellite arrays have a nested structure of layered expansions (Fig. 1), with CENPA usually occupying the central most homogeneous and youngest HOR, flanked successively by more divergent HORs and divergent monomeric α-satellites in the pericentromere (Altemose et al. 2022). The active HORs range in size from 340 kb to 4.8 Mb and have high levels of CG methylation, but CENPA occupies only a few hundred kb of the HOR surrounding a “centromere dip region” of low CG methylation. These core centromere dip regions have a characteristic “dichromatin” structure of densely compacted chromatin with frequent dinucleosome footprints interspersed with 6–28 accessible chromatin patches, that may correspond to the number of kinetochore microtubules (Dubocanin et al. 2025). The centromere dip regions are proposed to anchor the kinetochores by facilitating the binding of CENPB to the unmethylated CENPB box that is usually present in every other monomer (Parl 2024), which binding is reduced if the CENPB box is methylated (Tanaka et al. 2005a). CENPB interacts with and stabilizes CENPA and CENPC (Fachinetti et al. 2015). Chromosomes that lack CENPB boxes, such as the Y chromosome and neocentromeres, have higher rates of mis-segregation.

CENPB can dimerize and dimers bind adjacent CENPB boxes to form DNA loops, and to help position nucleosomes (Chardon et al. 2022; Dubocanin et al. 2025; Tanaka et al. 2005b; Yoda et al. 1998) These loops compact centromeric DNA, which might be important for CENPA loading or resisting anaphase forces on the kinetochore (Chardon et al. 2022). CENPC binds to CENPA and DNA and serves as a scaffold for building the kinetochore (Pesenti et al. 2022; Petrovic et al. 2016; Screpanti et al. 2011; Yatskevich et al. 2022). It can also dimerize (Chik et al. 2019; Cohen et al. 2008; Verma & Surolia 2014; Xiao et al. 2017), or in vertebrates oligomerize (Hara et al. 2023; Trazzi et al. 2009), to compact the centromere/kinetochore.

In the model plant *Arabidopsis thaliana* CENH3 similarly occupies the most homogeneous arrays of the 178 bp satellite *CEN180* (Naish et al. 2021). HORs of *CEN180* mostly have 2 or 3 monomers and are chromosome-specific, and show evidence of intrachromosomal homogenization through non-allelic gene conversion (Dong et al. 2025). *Arabidopsis* lacks a CENPB homolog and the centromere is heavily methylated at CG sites, but is less methylated at CHG and CHH sites than the flanking pericentric regions enriched in H3K9me2 (Naish et al. 2021). This is unsurprising since H3K9 methylation and non-CG DNA methylation are interdependent in *Arabidopsis* (Stroud et al. 2014). Since the most homogeneous HORs that support kinetochore formation in both animals and seed plants are also the youngest repeats of a family of rapidly evolving repeats, what drives this rapid satellite evolution?

## Centromere drive

**Fig. 2:**
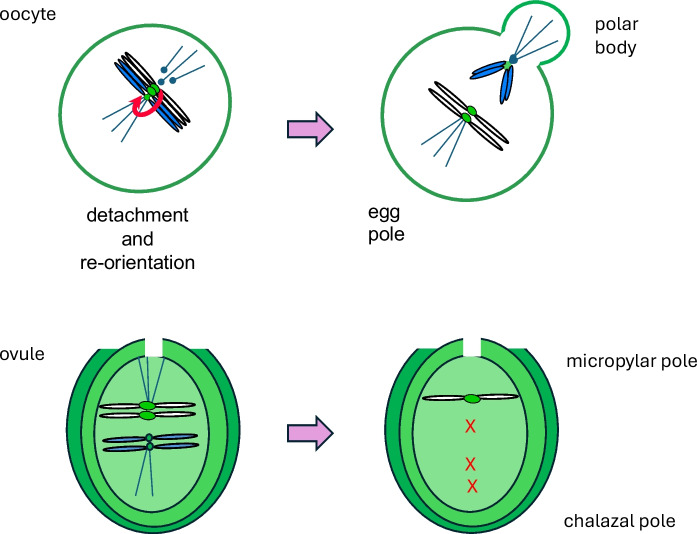
**Centromere drive** In mouse oocytes, stronger (typically larger) centromeres can more readily detach from microtubules and re-orient at the cortical pole so that they segregate preferentially to the egg pole where they are transmitted to the next generation (top). In ovules of monkeyflower and other flowering plants, meiosis in the megaspore mother cell favors the bivalent near the micropylar pole for inclusion in the surviving megaspore (bottom). How the stronger centromere orients preferentially to the micropylar pole is currently unknown

A study of CENPA (Cid) in *Drosophila melanogaster* and *D. simulans* surprisingly revealed that CENPA is evolving rapidly under positive selection, despite its highly conserved kinetochore function (Malik & Henikoff 2001). To explain this apparent paradox, the centromere drive model was proposed (Henikoff et al. 2001) In this model (Fig. 2), centromere variants can compete in the asymmetric reductional division of female meiosis I for inclusion in the egg (or megaspore in seed plants) rather than in the polar body. If a satellite expansion or other centromere variant results in the recruitment of more CENPA and/or other kinetochore proteins that can preferentially orient the centromere to enter the egg, such a ‘stronger’ centromere will be favored for transmission to the next generation. Such biased transmission should lead to rapid expansion and turnover of centromeric satellites. In symmetric male meiosis, however, it was hypothesized that unequal centromere strength could lead to checkpoint arrest or non-disjunction, and thus lead to positive selection for factors that suppress centromere drive, of which changes to CENPA, CENPC, and/or other kinetochore proteins are obvious candidates. In a second study, minor groove binding motifs were found to have evolved recurrently in the tails of CENPAs in *Drosophila*, mouse, and human, suggesting direct adaptation to centromeric DNA (Malik et al. 2002). Thus the rapid evolution of CENPA was proposed to be a response to the rapid evolution of competing centromere variants.

In the 24 years since the centromere drive model was proposed, considerable evidence supporting it has accumulated, reviewed in (Searle & Pardo-Manuel de Villena 2024; Talbert & Henikoff 2022). The first supporting evidence came from human Robertsonian translocations, in which two acrocentric chromosomes are combined into a metacentric chromosome often with a dicentric structure that has more microtubule-binding sites (Daniel 2002). Such translocations are preferentially inherited over their acrocentric counterparts through meiosis in female carriers (Pardo-Manuel de Villena & Sapienza 2001b), and have reduced fertility in male carriers (Daniel 2002), suggesting differences in centromere strength may underlie both phenomena, as predicted in the centromere drive model. That kinetochores might have different requirements in females and males is supported by the occurrence of sex-specific functionally diverged CENPA paralogs in the male and female germlines of fruit flies and mosquitos (Kursel & Malik 2017; Kursel et al. 2021, 2020) Examining CENPA/CENH3 evolution in 17 eukaryotic clades, positively selected branches were found more frequently in seed plants and animals, which have asymmetric meiosis, than in clades with symmetric meiosis (ferns, bryophytes, fungi, and apicomplexans), also as predicted by the centromere drive model (Zedek & Bures 2016). This suggests that centromere drive is widespread in seed plants and animals.

In the monkeyflower *Mimulus guttatus*, a driving allele *D* on chromosome *11* with a large expansion of centromeric satellite shows extremely biased transmission through female meiosis but not through male meiosis in crosses to the sibling species *M. nasutus* (Finseth et al. 2021; Fishman & Kelly 2015; Fishman & Saunders 2008; Fishman & Willis 2005). *D* heterozygotes did not show decreased pollen viability, but *DD* homozygotes have deleterious effects on both male pollen viability and female seed set, indicating that these linked deleterious recessive effects, rather than centromere strength differences in heterozygous male meiosis, probably keep *D* from coming to fixation in the population (Fishman & Kelly 2015; Fishman & Saunders 2008). Fishman & Kelly (2015) argued that heterozygous fitness costs are unlikely to result in antagonistic evolution, while homozygous costs can prevent fixation of drivers and allow time and selection pressure for suppressors to arise and spread in populations. In conspecific crosses, more modest *D* transmission bias was found against a chromosome *11* allele that lacks the satellite expansion, suggesting co-evolved suppression of drive within *M. guttatus*. At least part of this suppression is attributable to the chromosome *14* region that contains CENH3A, one of two CENH3 paralogs in *M. guttatus* (Finseth et al. 2015, 2021), suggesting that mutations in CENH3/CENPA can suppress drive, a key motivation of the centromere drive model. Both *D* and the *CENH3A* locus show indications of a selective sweep ~ 1000 years ago. Given that the origin and spread of a new CENH3A allele would likely require considerable time, Finseth et al (2021) argued that the suppressing CENH3A allele was likely pre-existing in the population and was able to spread because deleterious homozygous recessive fitness costs prevented fixation of *D*.

Differences in centromere strength and re-orientation of chromosomes on the asymmetric female meiotic spindle to bias segregation have been documented in mice. In hybrids between *Mus musculus* mouse strains differing in the amount of the centromeric minor satellite, chromosomes that have more minor satellite can load more CENPA, CENPB, CENPC, and more of the microtubule-attaching outer kinetochore protein HEC1 than their counterparts, and are favored for transmission into the egg in meiosis I (Chmatal et al. 2014; Iwata-Otsubo et al. 2017), even though in one hybrid the favored centromere with more minor satellite had less of the outer kinetochore protein SPC24 (Wu et al. 2018). The oocyte meiotic spindle is differentially tyrosinated on alpha tubulin at the cortical pole, which destabilizes microtubules at the cortical pole relative to the egg pole (Akera et al. 2017). Stronger centromeres undergo preferential detachment of microtubules at the cortical pole and subsequent re-orientation to the egg pole because they have more of the kinetochore protein BUB1 (budding uninhibited by benzimidazoles 1) kinase, which phosphorylates more H2ApT121, which recruits more SGO2 (Shugoshin 2) to pericentromeres where it serves as a scaffold for more MCAK (mitotic centromere-associated kinesin) and more Aurora B kinase (Akera et al. 2019). Aurora B kinase is essential for detachment of microtubules, and MCAK preferentially depolymerizes tyrosinated microtubules, enabling stronger centromeres to more readily detach at the cortical pole and re-orient to the egg pole. The role of pericentric MCAK in detachment and re-orientation suggests that the pericentromere is also important in modulating centromere drive, including non-kinetochore proteins that can affect the process of microtubule detachment and re-orientation without necessarily changing kinetochore size (Kumon et al. 2021). Positive selection has been identified in more than 15 proteins that might modulate biased segregation at female meiosis I, including kinetochore components, heterochromatin proteins, condensins, cohesins, and spindle proteins (Talbert & Henikoff 2022).

## Drive and sequence (in)dependence

Additional insight into the rapid evolution of kinetochore proteins comes from a recent study of the DNA-binding kinetochore protein CENPT in mice (Dudka, Nguyen, et al., 2025b). Adaptive evolution of CENPT was found to reduce its centromere binding, but independently of centromeric DNA sequence, indicating that it was not evolving in a direct sequence-specific response to changing DNA sequences. Rather, selected protein residues reduced the efficiency of its binding to its partner CENPW, resulting in less loading of the CENPT/W dimer at centromeres. This improved oocyte survival, suggesting that the fixation of a driving allele may have impacted oocyte fitness, similar to the effects of the *D* locus in *Mimulus*, and raising the possibility that centromere-associated adaptive evolution may frequently act to ameliorate deleterious effects that accompany the rapid fixation of driving centromere alleles, rather than to suppress biased segregation while the allele is still rapidly driving. How reduced binding of the CENPT/W dimer improves oocyte survival is not clear, but Dudka et al. have proposed that a fixed driven centromere with too many microtubule binding sites may disrupt the balance of microtubule attachment sites and microtubule destabilizing activity, negatively impacting kinetochore assembly at all centromeres, and thereby reducing oocyte survival and exerting selection pressure for kinetochore innovation.

Independence of DNA sequence may also apply to CENPC, which is under positive selection in both mammals and flowering plants (Talbert et al. 2004) but binds DNA non-specifically (Politi et al. 2002). CENPC targets CENH3/CENPA through its conserved CENPC motif, and in mammals also through the central domain, both of which bind CENPA nucleosomes (Kato et al. 2013). In *Arabidopsis*, the DNA binding regions flanking the CENPC motif are also required for stable centromeric localization (Yalagapati et al. 2025). The CENH3 licensing factor αKNL2 similarly requires both a CENPC-like motif (CENPC-k) and flanking regions that bind DNA non-specifically to stably target the centromere. The independence of DNA sequence may help preserve kinetochore function in the face of rapidly changing satellites.

In contrast, CENPB specifically binds the CENPB box. CENPB is a domesticated Tigger transposase (Kipling & Warburton 1997) that is non-essential in mice (Kapoor et al. 1998) though nevertheless widely conserved in mammals (Gamba & Fachinetti 2020). CENPB boxes, however, are only found in centromeres of a subset of mammals, suggesting centromeres with CENPB boxes have co-opted CENPB to augment existing centromere function. The absence of CENPB boxes on the Y chromosome suggests that CENPB box-containing α-satellites have spread through centromeres by centromere drive in females, perhaps to gain an advantage in transmission by stabilizing the kinetochore (Gamba & Fachinetti 2020; Marshall & Choo 2012). Indeed, the spread of CENPB boxes has happened independently three times in new world monkeys (Thongchum et al. 2020). Conversely, in horse satellite centromeres, ancestral CENPB boxes have been largely lost as they have been replaced with a shorter derivative satellite (Cappelletti et al. 2025).

## Drive, karyotype evolution, and speciation

In exceptional cases, centromere drive occurs in the absence of symmetric male meiosis. All mating types of the ciliate *Tetrahymena thermophila* undergo asymmetric meiosis with no contrasting symmetric meiosis. No *Tetrahymena* centromere sequences are as-yet fully assembled, but the putative centromere regions are unusually long at 5–10 Mb of repeats and transposons that together comprise 25% of the micronuclear genome, suggesting runaway centromere drive (Hamilton et al. 2016). There is no indication of positive selection on the *Tetrahymena* CENPA homolog CNP1 (Elde et al. 2011). Ants have haploid males so there is no male meiosis. In the fire ant *Solenopsis invicta*, runaway centromere drive in females has been proposed to explain the exceptionally long centromeric satellite arrays that make up on average a third of each chromosome (Huang et al. 2016). In the fungus-farming ant *Trachymyrmex holmgreni*, variation between populations in karyotype length seems to be primarily due to variations in centromere length, with more recent populations having significantly expanded centromeres (Cardoso et al. 2018). No populations were observed with polymorphic karyotype lengths, raising the possibility that no hybrid populations exist and centromere length may act as a reproductive barrier. Ant species vary widely in chromosome numbers, sometimes even within a species, and undergo chromosome fissions and fusions (Cardoso & Cristiano 2025). There is a positive correlation between the rate of genome breakpoints and species richness (Vizueta et al. 2025). Whether runaway centromere drive and centromere breaks contribute to this wide variation and species diversity is currently unknown.

Besides contributing to genome size solely by satellite expansion, a larger centromere with a larger kinetochore can presumably pull a larger chromosome, potentially accommodating gene duplication, non-centromeric tandem repeat expansion, and transposon invasion. Kinetochore size is correlated with genome size (Plačková et al. 2021; Zhang & Dawe 2012) and with chromosome size (Irvine et al. 2004; Plačková et al. 2022), suggesting that centromere drive may exercise control over both.

Centromere drive is known to be involved in fixing chromosome rearrangements, especially Robertsonian translocations and probably tandem fusions, reviewed in (Searle & Pardo-Manuel de Villena 2024). Such rearrangements present reproductive barriers that likely contribute to speciation (Capanna et al. 1976; Everett et al. 1996; Gropp et al. 1972; Hauffe & Searle 1998; Wallace et al. 2002). Karyotypes of 1170 species of mammals are biased toward mostly acrocentric or mostly metacentric, which might be explained if the polarity of the meiotic spindle in favoring acrocentric or metacentric chromosomes can switch during evolution (Blackmon et al. 2019; Pardo-Manuel de Villena & Sapienza 2001a). A similar karyotype distribution in actinopterygian fishes has also been attributed to centromere drive (Molina et al. 2014).

Plants undergo repeated rounds of whole genome duplication and subsequent postpolyploid diploidization, and this diploidization occurs more efficiently in plants with asymmetric meiosis, leading to lower chromosome numbers and often somewhat smaller chromosomes than in plants with symmetric meiosis (Kinosian & Barker 2025). It is proposed that this is at least in part a consequence of centromere drive, which can fix chromosome fusions thereby reducing chromosome numbers and potentially deleting redundant loci. Known fusions reducing chromosome number include Robertsonian translocations, end-to-end fusions with loss of one centromere, and nested fusions with one chromosome inserted into a pericentric break of another chromosome (Mayrose & Lysak 2021). Consistent with a role for centromere drive, seed plants show a faster rate of chromosome size evolution and a higher frequency of positive selection on CENH3 than plants with symmetric meiosis (Plačková et al. 2024).

Rapid divergence of satellites and kinetochore proteins can lead to incompatibilities that may also serve as reproductive barriers. In *Arabidopsis*, CENH3s that are from related species or with N-terminal tails that are divergently engineered can function fully in mitosis and vegetative growth, but when transmitted maternally in crosses with wildtype pollen, they are actively removed around egg maturation and the maternal chromosomes may be lost, generating haploids, with obvious implications for hybrid incompatibility (Marimuthu et al. 2021). Haploids are of great value to plant breeders, who can double them to quickly homozygose desired traits, and CENH3-based haploid induction systems have been applied to several crop species (Bortiri et al. 2024; Kaur et al. 2023; Lv et al. 2020; Manape et al. 2024; Musazade et al. 2025; Wang et al. 2024; Yang et al. 2025).

Several hybrid incompatibility genes encode chromatin proteins that interact with the pericentromere, which is also subject to satellite expansions and contractions and is rapidly evolving. Because meiotic recombination is typically suppressed in both the centromere and pericentromere (Nambiar & Smith 2016), pericentric satellite variants can be fixed along with their linked driving centromere, and can incur fitness costs if they are not packaged correctly. Satellite packaging proteins are often re-purposed from other uses. Some bind to specific satellite shapes (Dudka et al. 2025a), while others bind to specific DNA sequences present in both euchromatin and heterochromatin (Gaskill et al. 2023; Platero et al. 1998; Torok et al. 2000). The OdsH protein of *Drosophila mauritiana* causes hybrid male sterility when introgressed into *D. simulans* (Bayes & Malik 2009)*.* OdsH is a paralog of a transcription factor, and OdsH from both species binds to heterochromatin on chromosomes X and 4, but the divergent OdsH from *D. mauritiana* also is enriched on the Y chromosome of *D. simulans* males, decondensing it and causing hybrid inviability, whereas it shows only limited binding to the *D. mauritiana* Y chromosome.

In *D. melanogaster*, the 359 bp repeat comprises 11 Mb adjacent to the X chromosome centromere and is absent in the sibling species *D. simulans* (de Lima et al. 2020; Ferree & Barbash 2009; Lohe et al. 1993). Both topoisomerase II (topo II) and the MH (Spartan in humans) protein are required to correctly package the 359 bp repeat, and both are under positive selection (Brand & Levine 2022). The MH protein from *D. simulans* acts as a poison to this process, causing DNA damage and cell death when expressed in *D. melanogaster* ovaries. Spartan is a DNA repair protein that removes DNA–protein crosslinks, and it is hypothesized that the *D simulans* MH protein over-zealously removes topo II crosslinked to the 359 bp repeat, since the hybrid defect can be alleviated by deleting the 359 bp repeat or overexpressing topo II. In this scenario, MH is co-evolving with topo II and only indirectly with the 359 bp repeat but nevertheless results in hybrid incompatibility.

## Break-induced replication and satellite expansion

Centromere drive can favor transmitting centromeric satellite expansions over smaller satellite arrays (Chmatal et al. 2014; Finseth et al. 2021; Iwata-Otsubo et al. 2017), but how do expanded variants arise? Several non-mutually exclusive mechanisms have been proposed, including unequal homologous exchange (Smith 1974); gene conversion (Dover 1982, 2002); excision, rolling circle replication and re-integration of extrachromosomal circles (Cohen & Segal 2009); and break-induced replication (BIR) (Rice 2020a).

Unequal (out-of register) exchange between satellites on sister chromatids was first thought to produce duplications and deletions of satellites equally, but the discovery of the single-stranded annealing pathway (Lin et al. 1984) which repairs double-strand breaks (DSBs) by annealing homologous sequences and deleting the sequences between them, suggests a bias for satellite contraction rather than expansion over time. It is also unclear why this process would result in nested HORs in centromeres (Fig. 1). Gene conversion can homogenize repeats and could potentially expand or contract repeats, but probably mostly on a scale of less than 500 bp in humans (Masaki & Browning 2025) and ~ 125 bp in *Arabidopsis* centromeres (Dong et al. 2025). Extrachromosomal circles commonly arise from tandem arrays presumably by intrachromosomal crossover (Cohen & Segal 2009), and provide an attractive model for how satellites can spread to new chromosomes, but re-integration is likely to be much rarer than excision, suggesting long-term loss of repeats would be favored. In contrast, BIR occurs when an obstacle to replication causes fork collapse and generates a one-sided DSB, in which the free end gets resected and can then anneal out-of-register to similar repeats on its sister chromatid to generate duplications or deletions of one or more sets of tandem repeats (Rice 2020b, 2020c). In yeast rDNA repeats, BIR favors expansions over contractions (Kobayashi 2014), possibly because DNA ahead of the fork is overtwisted while DNA behind the fork is relaxed and more accessible. Centromeres are known hotspots for DNA breakage (Saayman et al. 2023; Scelfo & Fachinetti 2023), so could BIR account for centromere expansions?

**Fig. 3:**
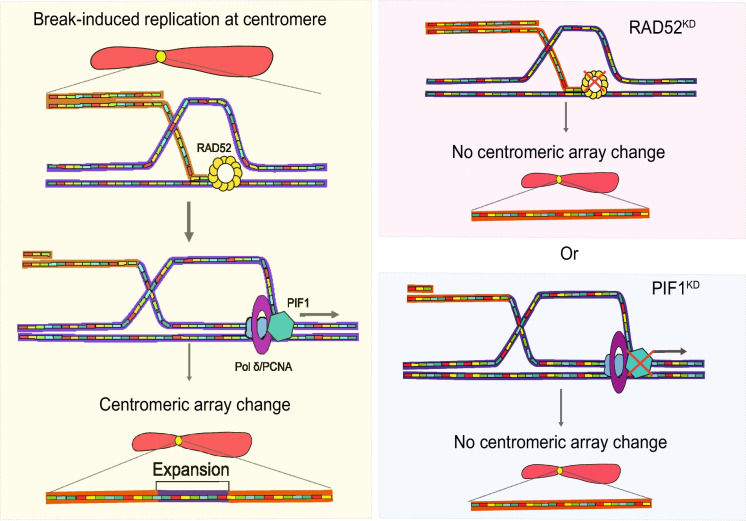
**Break-induced replication (BIR) in satellite centromeres** When a replication fork stalls one strand may be cleaved to generate a single-ended double strand break, which can be resected so the 3’ end can invade the sister chromatid (left). RAD52 promotes annealing, then polymerase δ and PCNA (proliferating cell nuclear antigen) begin synthesis with the aid of the PIF1 helicase. If the annealing takes place out of register in the D11Z1 HOR array in U2OS cells, it can lead to expansions or contractions. If RAD52 or PIF1 are knocked down, no array change occurs (right). Figure reprinted with permission from (Showman et al. 2024)

To test the BIR model (Fig. 3), copy numbers of the D11Z1 HOR were determined by digital droplet PCR at intervals of ~ 20 cell generations in subclones of the pediatric osteosarcoma cell line U2OS (Showman et al. 2024). U2OS cells utilize BIR to undergo Alternative Lengthening of Telomeres (Zhang et al. 2019), so they may form a sensitized system for detecting BIR-mediated centromere expansions. Both increases of copy number by as much as 43% relative to parental cells and decreases occurred in ~ 20 cell generations in ~ 42% of subclones, with expansions more frequent than contractions (Showman et al. 2024). Knockdowns of the strand-annealing protein *RAD52* (Sotiriou et al. 2016) and the conserved *PIF1* helicase (Wilson et al. 2013), which rescues stalled forks (Williams et al. 2023), prevented all expansions and contractions over 20 cell generations (Showman et al. 2024). Though RAD52 is required for both unequal exchange and BIR, PIF1 is required in BIR for the initiation of Pol δ-directed DNA synthesis after polymerase-helicase uncoupling upon stalling (Sotiriou et al. 2016; Williams et al. 2023; Wilson et al. 2013). These results indicate that notable satellite expansions and contractions can occur within 20 cell generations and depend primarily on *PIF1* and BIR. This suggests that BIR may underlie most of the variation we see among human individuals in satellite content (Logsdon et al. 2024) and may provide the allelic variation for centromere drive.

In *Arabidopsis* mutation accumulation lines, mutations in centromeres consist of precise in-frame indels of 1–31 satellite monomers, with insertions that arise from adjacent sequences occurring more frequently than deletions (Dong et al. 2025), consistent with BIR. Point mutations are 9 times more frequent in centromeres than in arms, but these are mostly accounted for by nearby intra-chromosomal non-allelic gene conversion tracks less than one monomer in length. Simulations demonstrated that these small scale indels and gene conversions can account for the formation of megabase-scale HOR structures.

BIR can efficiently expand existing tandem repeats, but how does the first tandem duplication occur and where do satellite sequences come from? In general we do not know, but a significant fraction of satellites derive from transposons (Ahmed & Liang 2012; Belyayev et al. 2020; Gong et al. 2012; Liu et al. 2024; Sharma et al. 2013; Tek et al. 2005; Veseljak et al. 2025), for reviews see (Meštrović et al. 2015; Zattera & Bruschi 2022). Some centrophilic transposons can target centromeric chromatin and accumulate there (Tsukahara et al. 2025). One way to produce a tandem duplication is if a transposon inserts into another copy of itself in the same orientation, which will produce two tandem duplications, one on either side of the insertion point. Either duplication could be expanded by BIR, and if it forms a suitable substrate for kinetochore formation, it may be preferentially transmitted through centromere drive. Whatever the initial size or sequence of a new satellite, it may converge over time to favor nucleosomal phasing. In sugarcane, longer younger transposon-derived centromeric satellites fail to phase centromeric nucleosomes, unlike an older, more diverse 137 bp centromeric satellite (Huang et al. 2021). In horses, the replacement of the 425 bp CENPB box-containing satellite with the 224 bp 37cen satellite derived from it (Cappelletti et al. 2025) might be an example of such convergence toward nucleosomal phasing.

## Centromere breaks and non-B-form DNA

Why do centromeres break? The BIR model originally proposed that the kinetochore itself caused replication forks to stall and break (Rice 2020a). However, depletion of CENPA during S phase but not other phases actually increased R loops and centromeric transcripts, resulting in unfinished replication, α-satellite recombination, and increased numbers of centromere breaks and centromeric translocations, with similar results upon depletion of the inner kinetochore proteins CENPC, CENPT or CENPW without reducing CENPA levels (Giunta & Funabiki 2017; Giunta et al. 2021). Depletion of the mitosis-specific outer kinetochore protein HEC1 caused mitotic mis-segregation but did not affect α-satellite recombination or rearrangements. Thus some feature of the inner kinetochore helps protect centromeres from breaks during replication.

A property of centromeres that does promote fork stalling and breakage is the presence of non-B-form DNA, DNA structures other than the standard Watson–Crick double helix including hairpins, cruciforms, G-quadruplexes (G4s) or intercalary motifs (i-motifs), R loops, and others (Duardo et al. 2023). Twenty-five years ago Koch noted that macaque α-satellite, yeast centromeres, and a human neocentromere on chromosome *10* all had predicted hairpin loops, which he suggested might form a structural code for functional centromeres (Koch 2000). More recently, dyad symmetries and other types of non-B DNA have been predicted in a wide variety of centromeres (Cappelletti et al. 2025; Chittoor & Giunta 2024; Despot-Slade et al. 2021; Hofstatter et al. 2022; Kasinathan & Henikoff 2018; Kuo et al. 2023; Liu et al. 2023, 2021; Patchigolla & Mellone 2022; Veseljak et al. 2025). In human cells, levels of non-B DNA detected by permanganate sequencing (Kouzine et al. 2017) correlated with CENPA occupancy and a quality score of CENPB boxes (Kasinathan & Henikoff 2018). In single molecule experiments, DNA from human centromeres *3*, *X*, and *Y* was found to form hairpins and other secondary structures whether it had CENPB boxes (*3*, *X*) or not (*Y*) (Chardon et al. 2022). As little as a single G4 or i-motif can cause fork stalling, helicase-polymerase uncoupling, and breakage, which can be repaired utilizing PIF1 (Williams et al. 2023). Non-B DNA is found not only in centromeric satellites, but seems to be a general feature of all satellites (Camacho et al. 2022). This suggests that repair by BIR of fork collapse caused by non-B DNA may have a causal role in generating all satellites.

Non-B DNA is also predicted in yeast point centromeres and human neocentromeres (Kasinathan & Henikoff 2018; Koch 2000), suggesting non-B DNA may have a specific role in centromere identity independent of a role in generating satellites. The human CENPA-loading chaperone HJURP (Holliday junction recognition protein) was originally identified because it interacted with mismatch repair proteins and could bind to four-way DNA junctions in vitro (Kato et al. 2007), raising the possibility that it may target CENPA to centromeric non-B structures. Alternatively, CENPA was reported to be deposited at DSBs (Zeitlin et al. 2009), in which case the association of centromeres with non-B DNA might be an instance of a more general association of centromeres with DSBs.

Non-B DNA is abundant in transposons, where it may have regulatory and targeting roles, reviewed in (Makova & Weissensteiner 2023). This may predispose the formation of satellites from transposons. Centromeres can also consist predominantly or entirely of transposons, especially in eukaryotes such as fungi with symmetric meiosis (Talbert & Henikoff 2020), possibly because large satellite arrays are unstable to recombinational removal in the absence of centromere drive, but transposon-rich centromeres are also found in some flowering plants and animals (Castellano et al. 2025; Courret et al. 2024; Houben et al. 2007; Liu et al. 2024; Ma et al. 2023; Nagaki et al. 2004; Schneider et al. 2016). Centrophilic retrotransposons have largely replaced satellites in some maize centromeres (Schneider et al. 2016), and centromeres in the *Drosophila simulans* subgroup suggest rapid cycles of invasion of centromeres by transposons alternating with expansions of centromeric satellites (Courret et al. 2024). In *D. melanogaster*, centromeres form around core retrotransposon islands, surrounded by simple satellites (Chang et al. 2019; Talbert et al. 2018). Non-B DNA was found on all centromeres: dyads were predicted only on the *Y* chromosome centromere that consists primarily of G2/Jockey3 retrotransposons, G4 enrichment was predicted on centromeres *X, 2*, and *4* (Patchigolla & Mellone 2022), while i-motifs were predicted for the *dodeca* satellite adjacent to centromere *3* (Garavis et al. 2015). Thus both satellites and transposons contribute to non-B DNA at centromeres, albeit to different types of non-B structures.

## Centromere breaks and cancer

Aneuploidy refers to the loss or gain of whole chromosomes or whole chromosome arms. Aneuploidies have been known to be present in cancers since 1890, when David Hansemann observed asymmetric mitoses in epithelial cancers but not in normal tissues (Hansemann, 1891). He illustrated chromatids away from the metaphase plate attached or unattached to the mitotic spindle as well as multipolar spindles, and considered that cancer was related to the chromosome losses he observed (Bignold et al. 2006). With the advent of whole genome sequencing and RNA sequencing (RNA-seq), copy number changes in whole chromosomes, whole arms, or partial gains and losses can be readily scored in cancers. Whole-arm aneuploidy is especially common in cancers (Ben-David & Amon 2020), and a centromere break is required to release a whole chromosome arm.

Are whole-arm aneuploidies important in cancer progression? Using RNA-seq data from 1298 meningiomas to assess gains and losses, whole-arm losses predicted rapid tumor recurrence (Zheng et al. 2025). To see if this result was restricted to meningiomas, WGS data from 10,522 patients in The Cancer Genome Atlas (TCGA) (Taylor et al. 2018) revealed an excess of whole-arm losses per patient (5.8) versus gains (4.2), and correlating aneuploidies with recurrence (Liu et al. 2018) gave a similar result to that for the meningiomas (Zheng et al. 2025). Thus whole-arm losses predict malignancy across human cancers.

**Fig. 4:**
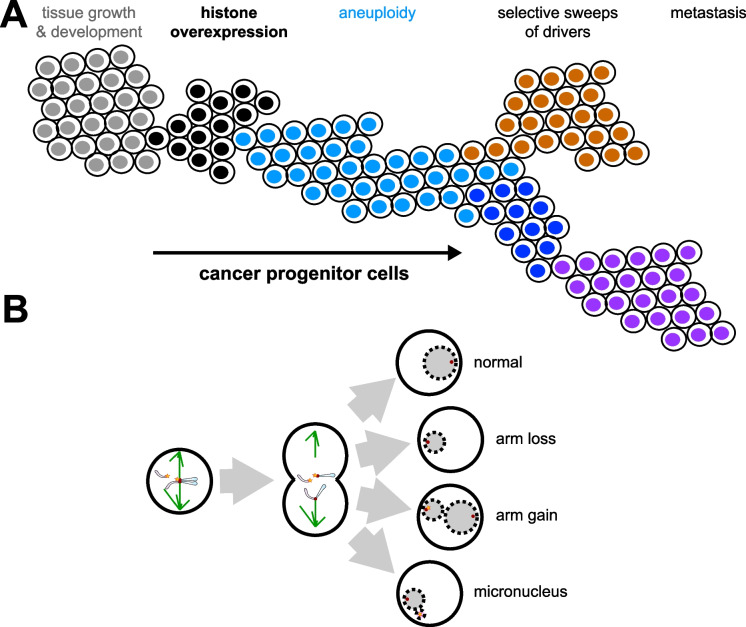
**Model for generation of centromere breaks leading to aneuploidy and tumor progression** (A) Cancer progression: Normal cells (grey) proliferate and differentiate to populate tissues, but aberrant induction of histone overexpression (black) promotes both hyperplasia and chromosome instability. Selection in hyperproliferating clones drives the frequencies of certain chromosomal abnormalities and the evolution of malignant cellular features (blue, brown, dark blue, purple). (B) Defective centromeres compromised by centromeric histone displacement will break, leading to widespread aneuploidy through whole chromosome arm loss, arm gain, and through whole chromosome loss. The occurrence of micronuclei by encapsulation of fragmented chromosome arms further stimulates chromosomal instability. Figure reprinted with permission from (Zheng et al. 2025)

Why are centromere breaks that generate whole arm aneuploidies so common in cancers? Another general feature of cancer cells is that RNA Polymerase II (RNAPII) is elevated relative to normal cells at the S-phase-dependent histone genes (Henikoff et al. 2025). Elevation of RNAPII at histone genes during S-phase is also a feature of pluripotent mouse embryonic stem cells (Mahat et al. 2024), and for cancer cells may be needed to decrease cell cycle duration and divide more rapidly (Chen et al. 2025). Overexpression of the 64 S-phase-specific histone genes, which are limiting for replication and cell proliferation, predicted aggressiveness in meningiomas and breast tumors, and also identified an excess of whole-arm chromosome losses relative to whole arm gains. This suggests that overexpression of S-phase histones may have a role in generating centromere breaks and the resultant whole arm aneuploidies. In addition to driving proliferation, overexpression of histones might be competing with CENPA nucleosomes during S-phase. Depletion of CENPA allows H3 nucleosomes to occupy centromeric chromatin (Blower et al. 2002), and during S-phase it causes increased breaks and rearrangements (Giunta et al. 2021). This suggests a model in which overexpressed S-phase histones displace centromeric nucleosomes, thereby causing breaks during replication that result in whole arm aneuploidies (Fig. 4). However, the traditional model is that aneuploidies are a result of segregation errors in mitosis.

These models can be distinguished by comparing metacentric and acrocentric chromosomes. Because their short arms have only dispensable content, a break in the centromere of an acrocentric chromosome is indistinguishable from a whole acrocentric chromosome. Thus if mitotic mis-segregation contributes significantly to aneuploidy, apparent acrocentric “whole-arm” aneuploids would be expected to be contributed by both centromere breaks and whole chromosome mis-segregations and to be more frequent than metacentric whole arm aneuploids, since the latter are contributed solely by centromere breaks. However, the frequency of whole-arm aneuploidy does not differ significantly between metacentrics and acrocentrics (Zheng et al. 2025), suggesting that there is no significant contribution of mitotic mis-segregation to aneuploidy, and that acrocentric whole-arm aneuploidy occurs largely as a consequence of centromere breakage during replication.

This raises the possibility of a general mechanism underlying diverse cancers in which overexpressed S-phase histones drive proliferation and promote centromere breaks, generating whole arm aneuploids. As S-phase histones reside in the histone locus body, are regulated by a unique transcription factor NPAT and the U7 small nuclear RNP, and produce the only transcripts to have a 3’ stem-loop with a stem-loop binding protein, they present several unique targets that could provide new general anti-cancer therapeutic options.

## Perspective

In the 40 years since the identification of CENPA, CENPB, and CENPC researchers have uncovered a surprising diversity of centromere and kinetochore structures, of which only satellite centromeres are discussed here, and revealed not only the complex architecture and mechanics of cell division but the unexpected roles of these proteins in genome integrity, genome evolution, speciation, plant breeding, and human disease. The observation of non-B DNA in centromeres is now frequently confirmed in new species and has contributed to the renewed interest in the roles of these structures throughout genomes, as well as research into their accommodation and repair during replication. Many open questions of satellite array expansion and contraction remain, including the generality of the BIR model in other cells and organisms, which other proposed mechanisms of satellite expansion and contraction might also apply and in which circumstances, what limits satellite expansions, and how satellites move from one chromosome to another. While the centromere drive model is now widely accepted, there is still much to learn about its occurrence in nature and how proteins respond to driving alleles or their legacy of satellite changes, hitchhiking effects, karyotype changes and speciation. Recent data on whole-arm aneuploidy in cancer require rethinking both its mechanism and roles, and its correlation with aggression and histone overexpression hold out the hope of a unifying theory of cancers and their treatment.

## Data Availability

No datasets were generated or analysed during the current study.

## Abbreviations

BIR: Break-induced replication – a repair process for collapsed replication forks that requires the PIF1 helicase
CENP: Centromere protein – one of several kinetochore proteins named with distinguishing letters, e.g. CENPA
CREST: Calcinosis, Raynaud’s phenomenon, esophageal dysmotility, sclerodactyly, telangiectasia – an auto-immune disorder which often produces anti-centromere antibodies
HOR: Higher order repeat – highly similar repeats composed of sets of dissimilar α-satellite monomers
